# Fast Response GaN Nanoscale Air Channel Diodes with Highly Stable 10 mA Output Current toward Wafer‐Scale Fabrication

**DOI:** 10.1002/advs.202206385

**Published:** 2023-04-20

**Authors:** Yazhou Wei, Feiliang Chen, Ruihan Huang, Jianpeng Zhao, Haiquan Zhao, Jiachao Wang, Mo Li, Jian Zhang

**Affiliations:** ^1^ School of Electronic Science and Engineering University of Electronic Science and Technology of China Chengdu 611731 China; ^2^ Yangtze Delta Region Institute University of Electronic Science and Technology of China Huzhou 313000 China

**Keywords:** fast response, GaN nanoscale air channel diodes, mA‐level output current, temperature‐dependence performance, wafer‐scale fabrication

## Abstract

Nanoscale air channel transistors (NACTs) have received significant attention due to their remarkable high‐frequency performance and high switching speed, which is enabled by the ballistic transport of electrons in sub‐100 nm air channels. Despite these advantages, NACTs are still limited by low currents and instability compared to solid‐state devices. GaN, with its low electron affinity, strong thermal and chemical stability, and high breakdown electric field, presents an appealing candidate as a field emission material. Here, a vertical GaN nanoscale air channel diode (NACD) with a 50 nm air channel is reported, fabricated by low‐cost IC‐compatible manufacturing technologies on a 2‐inch sapphire wafer. The device boasts a record field emission current of 11 mA at 10 V in the air and exhibits outstanding stability during cyclic, long‐term, and pulsed voltage testing. Additionally, it displays fast switching characteristics and good repeatability with a response time of fewer than 10 ns. Moreover, the temperature‐dependent performance of the device can guide the design of GaN NACTs for applications in extreme conditions. The research holds great promise for large current NACTs and will speed up their practical implementation.

## Graphical abstract

A vertical GaN nanodiode with a 50 nm air channel is reported, fabricated using IC‐compatible technologies, with a record field emission current of 11 mA@10 V. Notably, the device displays outstanding stability and fast switching characteristics with a sub‐10 ns response time. Additionally, the temperature‐dependent performance can guide the design of GaN NACTs for applications in extreme conditions.

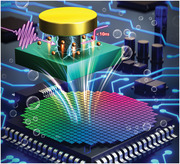

## 1 Introduction

Vacuum tubes, once the cornerstone of early electronics, have been nearly superseded by light, power‐efficient, low‐cost, and integrated solid‐state devices.^[^ ^1^ ^]^ However, the development of nano and micro‐scale manufacturing technologies has promoted a novel device named nanoscale air channel transistor (NACT),^[^ ^2^ ^]^ which combines the high‐frequency properties of vacuum electronics with the scalability of solid‐state devices.^[^ ^3^ ^]^ Because the length of the air channel in the NACT is close to or less than the mean free path (MFP) in air (≈68 nm),^[^ ^4^ ^]^ scattering‐free or ballistic transport of electrons in the air channel is possible without requiring vacuum conditions. This nanoscale air channel also enables the NACT to operate with features of high frequency and fast response under harsh environments, making it ideal for millimeter‐wave/terahertz applications in aerospace or nuclear scenarios.^[^ ^2^, ^5^ ^]^

Because of their well‐established fabrication techniques, initial attempts to create NACTs have largely concentrated on using Si^[^ ^2^, ^6^ ^]^ and metals^[^ ^5^, ^7^ ^]^ as the cathodes. In 2012, NASA and the University of Pittsburgh achieved Si‐based NACTs with planar and vertical architectures with photoresist ashing and focused ion beam (FIB), respectively.^[^ ^2a,b^ ^]^ Following that, several endeavors were reported. Some efforts focused on aggressively scaling down the air channel. For example, the self‐assembly of polystyrene nanospheres was applied to build a metal‐based nanodiode with a sub‐10 nm air channel, resulting in a device with a low turn‐on voltage of 0.7 V.^[^ ^8^ ^]^ Other work mainly emphasized improving device structure, such as utilizing a tip cathode or introducing alternative gate types to increase the device's performance. For instance, a TiN‐based nanodiode with a bow‐tie structure fabricated by electron beam lithography (EBL) with a 19 nm air channel obtained a ten times field enhancement factor.^[^ ^9^ ^]^ Additionally, back‐gate,^[^ ^5a^ ^]^ side‐gate,^[^ ^7^ ^]^ double‐gate,^[^ ^10^ ^]^ surround‐gate,^[^ ^2c^ ^]^ and Fin‐gate^[^ ^11^ ^]^ structures, as well as supporting theoretical research, have been proposed sequentially to construct NACTs. Several studies have also explored the behaviors of NACTs under harsh conditions, such as irradiation,^[^ ^2^, ^5^ ^]^ revealing their high potential for applications in extreme situations such as aerospace.

Despite the advances, the practical development of NACTs is still restricted by the low currents (usually in the µA‐level) caused by the limited emission area of the cathode, as well as the complex device preparation processes. Furthermore, the stability of tip structures is also significantly compromised due to the low breakdown field of the cathode material and the extremely concentrated electric field at the cathode tip.^[^ ^12^ ^]^ GaN is a wide‐bandgap material with a lower electron affinity (2.7–3.3 eV) compared to Si and metals, which favors electron emission. Moreover, GaN possesses superior physical and chemical properties, such as a ten times higher breakdown field (3.3 MV cm^−1^) than Si, a high melting point (1700 °C),^[^ ^13^ ^]^ and excellent thermal conductivity, all of which are necessary for stable electron emission in harsh settings. Furthermore, due to the popularity of LEDs and RF power devices, GaN has more developed growth and fabrication techniques than other wide bandgap materials such as diamond and SiC.^[^ ^13^, ^14^ ^]^ As a result, GaN is an ideal candidate for the mass production of high‐performance integrated circuits NACTs. Recently, Zhao and co‐workers demonstrated a lateral GaN nanoscale air channel diode (NACD) with an air channel of 45 nm and a field emission current of 40 µA@3 V.^[^ ^15^ ^]^ Sandia National Laboratory reported a tip‐to‐edge GaN NACD with a channel of 30 nm created by EBL, with a field emission current of 457.1 nA@1 V, and no obvious performance degradation was observed with a current up to 10 µA.^[^ ^14b^ ^]^ Overall, research on GaN NACDs is very lacking.

In the previous study, we conducted a simulation to explore the relationship between the structure of GaN NACD and its performance.^[^ ^16^ ^]^ Here, we demonstrate a vertical GaN NACD fabricated by IC‐compatible techniques on a 2‐inch sapphire wafer with an air channel down to 50 nm for the first time. An output current of 0.522 mA@3 V, and 11 mA@10 V is achieved, which surpasses the results of previously reported NACDs. The effects of the anode size on GaN NACDs performance are also examined. The device structure, combined with the great thermal and chemical stability of GaN and the robust manufacturing processes, ensures excellent stability in cyclic, long‐term, and pulsed tests. It also has a fast response time of about 10 ns. Furthermore, the temperature‐dependent performance of GaN NACDs up to 400 °C is investigated, providing useful information for designing GaN NACDs for extreme environment applications in the future.

## 2 Results and Discussion

### 2.1 Electrical Performance and Operating Mechanisms of the GaN NACD

**Figure 1:**
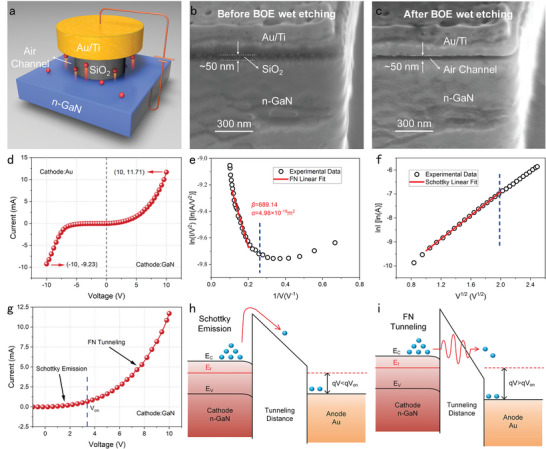
Device structure, electrical properties, and the operating mechanism of GaN NACD. a) Schematic structure of the GaN NACD and its external circuit. Devices C1–C4 with four sizes of Au anodes were fabricated with radii of 2546, 1273, 647, and 318 µm, respectively. b) SEM image of device section prepared by FIB cutting before BOE wet etching. c) SEM image of the device section after BOE wet etching, with a 50 nm high air channel, consistent with the thickness of the oxide layer. d) *I–V* characteristics of C1 device. e) FN linear fit of *I–V* data of the C1 device, with an FN tunneling turn‐on voltage (*V*_on_) of about 3.79 V. f) Schottky linear fit of *I–V* data of the C1 device in Richardson–Schottky coordinates (ln*I* versus *V* ^1/2^). g) Two operating mechanisms of GaN NACD: Schottky emission at *V* < *V*_on_ and FN tunneling at *V* ≥ *V*_on_. h,i) Energy band diagrams of GaN NACD with Schottky emission h) at *V* < *V*_on_ and FN tunneling i) at *V* ≥ *V*_on_. *E*_C_, *E*_F_, and *E*_V_ denote the conduction band, Fermi level, and valence band, respectively.

Generally, NACDs come in two structural types, vertical and planar, defined by the direction of electron transport. Planar structures provide a broad design space for device architecture, but expensive processing techniques such as EBL and FIB are required to fabricate the nanogaps,^[^ ^17^ ^]^ hence limiting their mass manufacturing. In contrast, the thickness of the oxide layer determines the length of the air channel in a vertical NACD, and the channel can be created by IC‐compatible methods, such as reactive ion etching (RIE)^[^ ^18^ ^]^ or buffered‐oxide etchant (BOE),^[^ ^19^ ^]^ allowing mass‐produced, integrated, low‐cost devices. Here we proposed a vertical GaN NACDs, the schematic diagram of the GaN NACD manufactured using IC‐compatible techniques, as shown in **Figure** **1**a. Devices C1 to C4 with Au electrodes of four radii of 2546, 1273, 647, and 318 µm were created. Detailed preparation procedures are available in the Methods and Supplementary Information. Scanning electron microscope (SEM) images of the device section before and after BOE wet etching are shown in Figures 1b,c. The BOE etching creates an air channel of 50 nm between the Au electrode and the GaN, which is consistent with the thickness of the oxide layer. Moreover, the extended BOE etching time and the significant voltage difference between the cathode and anode will worsen the electrode bending, similar to nanoelectromechanical switches,^[^ ^20^ ^]^ and ultimately lead to contact between the Au electrode and the GaN, which needs to be circumvented. More details on the stability of the devices under electrostatic forces can be found in Section 2.3 and in the Supporting Information.

In this paper, all experiments were conducted in the atmospheric environment. The measured *I–V* characteristic of the C1 device is shown Figure 1d, where the current increases exponentially with both positive and negative driving voltages. Electron transport in the air channel developed by BOE etching and the leakage test of the device is given in Figure S1, Supporting Information. The maximum output current obtained is 9.23 mA@−10 V and 11.71 mA@10 V for Au and GaN cathodes, respectively. Correspondingly, the *V*_100 µA_ of NACD is −2.7 and 1.1 V, respectively. Here, *V*_100 µA_ is defined as the voltage when the output current *I* is about 100 µA. The lower *V*_100 µA_ of the GaN cathode is most likely due to its lower electron affinity (<4.0 eV) than Au (5.1 eV), which favors electron emission. Despite having less free electrons in the conduction band than Au and the electrons emitted by GaN may not be entirely captured by the Au anode, GaN has a higher field emission current at 10 V than the Au cathode. This highlights the critical need for low work function materials as field emission cathodes.

In general, Fowler–Nordheim (FN) tunneling and Schottky emission are the two primary mechanisms of the electron emission performances of NACDs.^[^ ^5^, ^12^, ^21^ ^]^ The simplified linear FN equation explaining the relationship between the field emission current and anode voltage is given by Equation (1)^[^ ^22^ ^]^

(1) $$\ln\left(I/V^{2}\right)=\ln A-B/V$$

where *A* and *B* are the linear and exponential factors of the field emission current with values of *A* = 1.54×10^−6^ *β* ^2^ *φ* ^−1^d^−2^ *α* and *B* = 6.83×10^9^ *dφ* ^3/2^ *β^−1^*. Here *α* is the effective field emission area, *β* is the field enhancement factor, *φ* is the work function of the cathode (*φ*_Au_ = 5.1 eV, *φ*_GaN_ = 4.0 eV), and *d* is the length of the air channel. As is shown in Figure 1e, the data curve becomes linear from 3.79 V (V_on_), indicating that electron emission follows the FN tunneling mechanism. According to Equation (1), the fitting *α* is 4.98 × 10^−18^ m^2^ and *β* is 689.14 for the C1 device.

When the anode voltage is less than *V*_on_, the electron emission current follows the Schottky emission mechanism, which can be described below^[^ ^23^ ^]^

(2) $$\ln I\propto CV^{1/2}$$

where *C* is a constant related to the emitter shape, air channel length, and the temperature near the cathode. Details of the equations for FN tunneling and Schottky emission are provided in Supporting Information. If there is a linear relationship in Richardson–Schottky coordinates (ln*I* versus *V* ^1/2^), as seen in Figure 1f with a voltage less than *V*_on_, the electron emission is dominated by Schottky emission. Hence, GaN NACDs have two operating mechanisms, Schottky emission at low voltages and FN tunneling at higher voltages, as depicted in Figure 1g.

The energy band diagrams of Schottky emission and FN tunneling are shown in Figures 1h and 1i, respectively. When the applied voltage is less than *V*_on_, the vacuum level bends insignificantly and the interface barrier is too thick for electron tunneling, resulting in a longer tunneling distance for Schottky emission than FN tunneling. Due to thermal effects, only a small number of electrons can cross the GaN–air interface barrier in this situation, as seen in Figure 1h. When the anode voltage exceeds *V*_on_, the vacuum level bends downward more prominently due to the high‐intensity electric field at the surface, making the barrier thinner and lower for electrons tunneling. FN tunneling gradually becomes the primary mechanism of electron emission.

**Table** **1** compares the performance of our GaN NACD to that of previously reported devices. It is noted that the NACTs with sub‐MFP air channels exhibiting excellent field emission performance have been created using IC‐compatible methods. Among them, our device has a relatively low *V*_on_ and an outstanding field emission current of 11.7 mA@10 V, which is much higher than the previous reported NACTs^[^ ^10^ ^]^ (generally much less than 1 mA@10 V). There are three possible explanations for the high output current of our GaN NACD. To begin, due to its low electron affinity (2.7–3.3 eV), GaN is an ideal field emission material for electron emission. The electron affinity of GaN, in particular, can be reduced further by doping or altering the alloy component, with a minimum value of about 2.37 eV.^[^ ^24^ ^]^ Besides, when a high electric field is applied, the strong polarization within the GaN can be enhanced even more, creating a substantial bending of the energy band^[^ ^25^ ^]^ on the GaN surface and the accumulation of a large number of electrons near the bottom of the conduction band for tunneling. Finally, the flat cathode with considerable emission area also contributes to the improved performance of the GaN NACD. Our vertical design, combined with the IC‐compatible techniques, makes it possible to construct sub‐MFP air channels, which leads to a high electric field intensity at the cathode surface, promoting electron tunneling.

**Table 1:** Comparison of electrical performances of published NACTs

Device structure	Cathode material	Fabrication method	Channel length [nm]	Turn‐on voltage [V]	Current density [A cm^−2^]
Vertical diode^[^ ^19^ ^]^	Si	BOE	80	1.08	1.16
Planar triode^[^ ^2a^ ^]^	Si	Ashing technique	150	8.9^a)^	≈3.47×10^5^
Planar diode^[^ ^12a^ ^]^	Au	EBL & FIB	5	0.588	≈4.2×10^6^
Planar diode^[^ ^26^ ^]^	Metal	Nanosphere lithography	6.88	0.7	‐
Planar diode^[^ ^27^ ^]^	Ta	EBL	24	0.5	‐
Planar triode^[^ ^21^ ^]^	PdO	Electro‐forming process	80–90	5	‐
Vertical triode^[^ ^5b^ ^]^	SiC	RIE	200	≈10	‐
Planar diode^[^ ^15^ ^]^	AlGaN/GaN	BOE	45	2.3	9.41
Planar diode^[^ ^14b^ ^]^	GaN	EBL	30	0.24^b)^	≈1.47×10^4^
This work (C1)	GaN	BOE	50	3.79	2.44×10^2^

^a)^ The turn‐on voltage is defined as the voltage required to obtain an electric field of 1 V µm^−1^ around the tip;

^b)^ The turn‐on voltage is defined for emission current *I* ≥ 100 pA.

### 2.2 Anode‐Size Dependence of the Performance of GaN NACDs

For NACDs, the emission area is a crucial structural component responsible for the performance of the device. In prior work, Siwapon Srisonphan et al.^[^ ^2b^ ^]^ discovered that the field emission current of the vertical NACD was proportional to its circumference, indicating that electron emission occurs at the cathode's edge surface. As shown in Figure S2a, Supporting Information, finite element simulations (software: COMSOL Multiphysics 5.6) were performed to examine the electrical field distribution in the air channel and the electron emission behavior of the GaN surface. It can be observed that our device is a surface‐emission type, with the emission region being the part of the GaN surface toward the Au electrode. Therefore, the anode size has a significant effect on the field emission current of GaN. On the one hand, the anode structure influences the electric field distribution in the air channel and determines the probability of electrons tunneling on the GaN surface. On the other hand, the anode impacts the electron trajectory and the ratio of collected electrons. Both factors affect the output current. Until now, the effect of performance dependence on the anode has not been thoroughly examined. As a result, four devices C1 to C4 with different anode sizes were fabricated, with GaN–Au facing areas of 4800, 2400, 1200, and 600 µm^2^.

**Figure 2:**
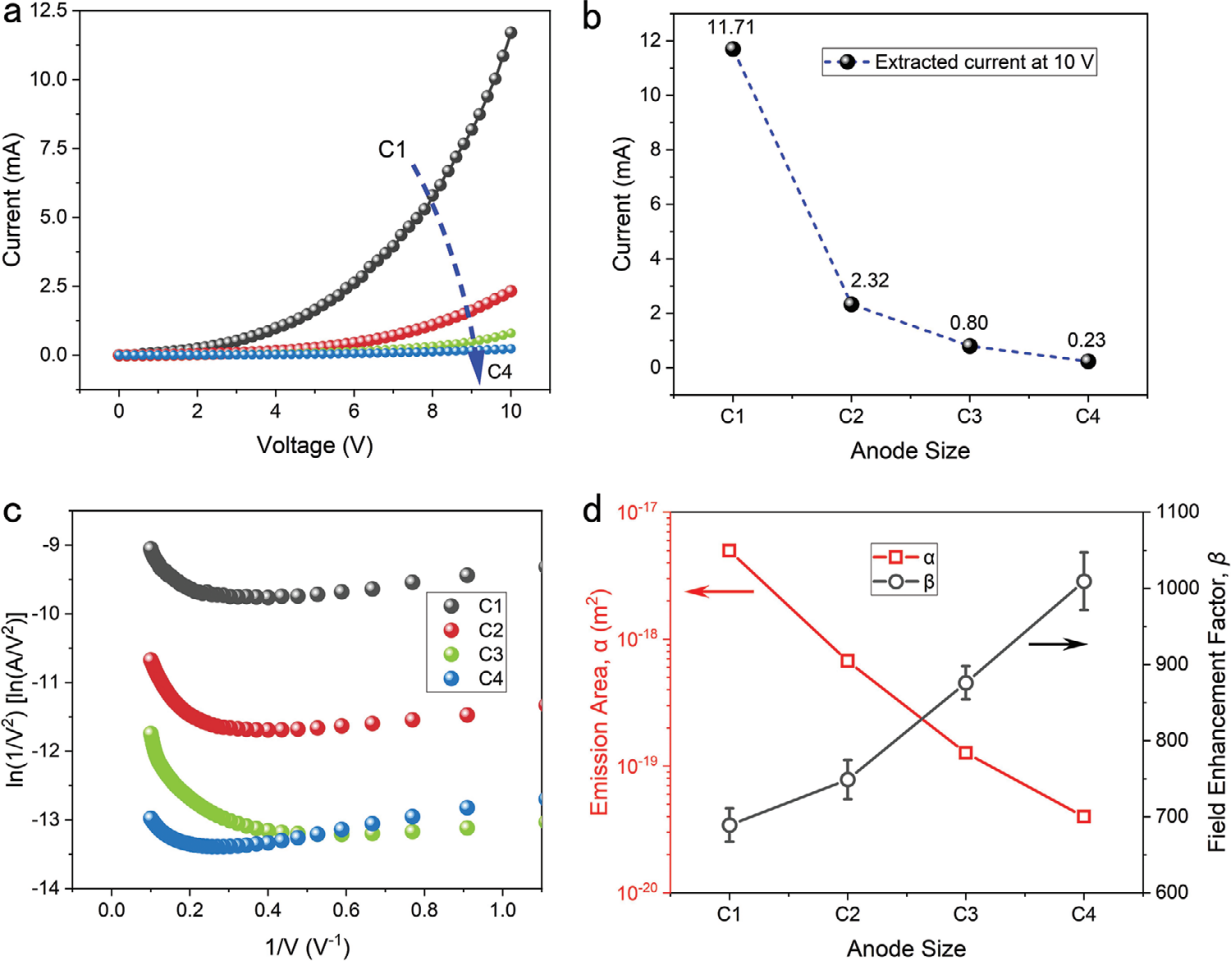
Electrical characteristics of GaN NACDs with four anode sizes. a) *I–V* characteristics of the four devices C1–C4. b) Extraction of the currents of C1–C4 at 10 V. c) Corresponding FN plots of C1–C4 with *V*_on_s of 3.79, 3.40, 3.51, and 3.80 V, respectively. d) Effective field emission area *α* and field enhancement factor *β* as a function of the anode size.

The measured *I–V* characteristics of the devices of C1 to C4 are plotted in **Figure** **2**a, where the emission currents grow exponentially with the applied voltages. The currents of C1 to C4 at 10 V are 11.71, 2.32, 0.80, and 0.23 mA, respectively, as shown in Figure 2b. All output currents are in the mA‐level, which is more than 2–3 orders of magnitude higher than that of the proposed NACDs (generally in µA‐level, as shown in Table 1). Devices with larger anodes have higher output currents due to increased electron emission area and probability of reception. The linearity of the ln(*I*/*V* ^2^) versus 1/*V* plots in Figure 2c confirms the FN tunneling mechanism of the devices C1 to C4 with corresponding *V*_on_ of 3.79 V, 3.40 V, 3.51 V, and 3.80 V, all close to 3.5 V. Figure S3, Supporting Information shows the SEM images of cross‐sections of the electrode edges of devices C2 to C4. It can be observed that these devices have nearly uniform air channels, which is attributed to the stable IC‐compatible manufacturing technologies.

The FN parameters *α* and *β* of the four devices are shown in Figure 2d. As seen, *α* decreases rapidly from 4.98×10^−18^ to 3.99×10^−20^ m^2^, whereas *β* grows linearly from 689 to 1009 as the anode size decreases. The increase of *β* with a smaller anode size may due to the electric field screening effect. For a field emission region with a limited area, the corner region has a stronger electric field and current density, while the central region presents relatively weak field electron emission.^[^ ^28^ ^]^ When the anode size increases, the GaN surface has a larger emission area, and the electric field shielding effect is more prominent, resulting in a smaller *β*. The larger Au–GaN facing area needs to increase the depth of the air channel achieved by prolonging the BOE etching time, which may cause the edge of the Au electrode to bend and contact the GaN. This can be solved by replacing the material with toughness or increasing the thickness of the Au electrode.

### 2.3 Stability Investigations of the GaN NACDs

**Figure 3:**
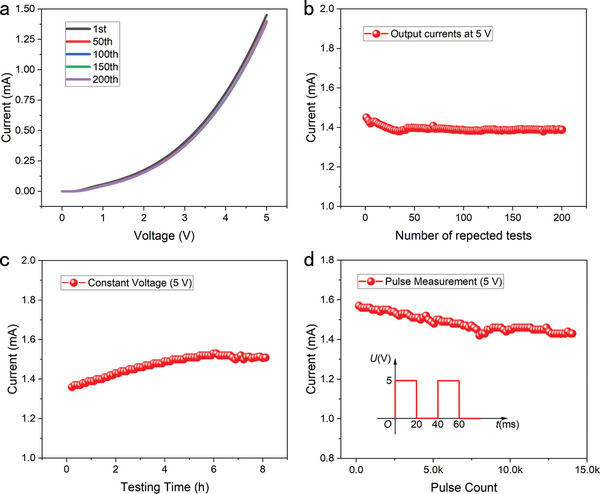
Stability tests of GaN NACD (C1) in the air. a) Output current of 200 cycles testing with drive voltage from 0 to 5 V. b) Output current at 5 V of the 200 cycles test. c) Long‐term (8 h) stability tests with a voltage of 5 V. d) Output current of 14 000 pulsed tests. The inset is the schematic diagram of the pulsed driving voltage (pulse width of 20 ms, duty cycle of 50%, peak voltage of 5 V).

For practical applications, the NACTs’ long‐term operation stability is a substantial hurdle. The performance of NACTs is susceptible to morphological damages induced by oxidation, electric field breakdown,^[^ ^29^ ^]^ ion bombardment,^[^ ^30^ ^]^ and Joule heating.^[^ ^31^ ^]^ For NACDs with tip‐type cathodes, the difficulties are particularly challenging. Here, three types of stability of the GaN NACD are examined. First, the device is conducted for cycle testing. As illustrated in **Figure** **3**a, device C1 is tested for 200 cycles from 0 to 5 V with very good reproducible performance. The current is compared for 200 cycles of testing at 5 V, as shown in Figure 3b, which drops gradually and eventually stabilizes. The degradation is only about 4.83%, from an initial value of 1.45 mA to the stabilized value of 1.38 mA, which is superior to that of the metal planar structure of 20% over 200 tests.^[^ ^12a^ ^]^ The decrease in current during the initial 50 tests may be induced by the impurity migration.^[^ ^19^ ^]^ There are modest oscillations in the following 150 tests that may be caused by the absorption and desorption of the molecules in the air.^[^ ^32^ ^]^

The long‐term stability of the GaN NACD is also investigated. The device of C1 is subjected to a constant voltage of 5 V for 8 h, and the current–time curve is shown in Figure 3c. With prolonged testing time, the output current increases by 11.76% from 1.36 mA to a stable value of 1.52 mA. In terms of long‐term stability, our device surpasses the previously reported planar‐structure GaN diode with a current decay of 40% within 8 h.^[^ ^14b^ ^]^ The minor increase in current may be due to the increase of the GaN surface temperature caused by the accumulation of Joule heat,^[^ ^21^ ^]^ which boosts the electron energy and enhances the probability of electron tunneling. Additionally, finite element simulations are performed to examine the deformation of the Au electrode under strong electric field. As demonstrated in Figure S2b, Supporting Information, the deformation value of the Au electrode under an electric field of 2×10^8^ V m^−1^ is only 0.036 nm without significant bending. It means our device can operate stably for long‐term periods under strong electric fields.

In some real‐world applications, NACDs will operate in AC mode for high‐speed or high‐frequency scenarios. As a result, the device of C1 is treated to 14 000 square pulses with a peak voltage of 5 V, a pulse length of 20 ms, and a duty cycle of 50%. As shown in Figure 3d, the output current decreases gradually during the first 8000 pulses before leveling off at a reduction of about 8.28%. The consistent output of our device under AC signal highlights the enormous application potential of NACDs in future logic circuits. The high stability of our device may be attributed to its burr‐free flat architectures, robust IC‐compatible techniques, and the superb physical and chemical properties of GaN. Thereby, severe current degradation caused by surface oxidation, tip sharpness loss, electric field breakdown, ion bombardment, and other factors is effectively averted during device operation.

### 2.4 Temporal Response Measurement

**Figure 4:**
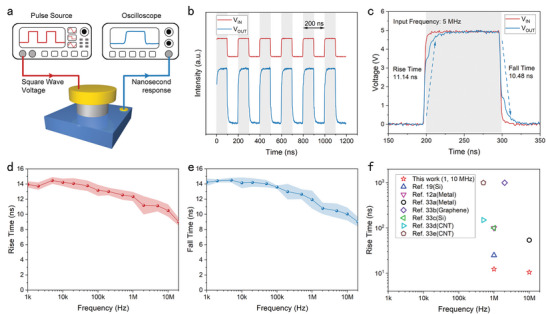
Temporal response measurements of the GaN NACDs. a) Schematic of the experimental set‐up of the response time test with the GaN NACDs. The response speed of the devices is acquired by an oscilloscope. The input signal of a nanosecond pulse square wave is provided by a signal generator with an amplitude of 5 V, a rise time of 3 ns, and a duty cycle of 50%. b) The output waveform of device C1 demonstrates a high switching frequency at 5 MHz. c) A single cycle of the 5 MHz input waveform and the output waveform. The output shows a rise time of 11.14 ns and a fall time of 10.48 ns. d,e) The rise time (d) and the fall time (e) of the output with various input frequencies from 1 kHz to 20 MHz. f) Comparison of the response times of representative reported NACTs made of different materials (including Si, metals, nanotubes, and graphene). Our work shows the fastest response time, down to 9.02 ns.

The response time of the NACDs is one of the key performances for high‐speed applications, demonstrating the capacity to track fast signals. However, FN‐tunneling‐based NACTs still lack experimental investigations on their temporal response performance. Here we evaluated the response speed of our proposed GaN NACDs. **Figure** **4**a depicts the experimental setup for measuring response time. A nanosecond pulse signal source drives the device under test, which is connected to an oscilloscope for acquiring the response speed. In Figure 4b, the switching frequency of device C1 with a 5 MHz input signal at 5 V is illustrated. The output waveforms of our GaN NACDs exhibit exceptional fast‐switching characteristics with high repeatability.

Figure 4c shows a single cycle of the input and output signals of the device. We can see the GaN NACD has a rise time of 11.14 ns and a fall time of 10.48 ns at 5 MHz input. Here, the rise(fall) time is defined as the time required for the output signal amplitude to grow(drop) from 10%(90%) to 90%(10%) of its maximum value. The fast switching speed reveals the substantial promise of the device for high‐speed electronic systems. Then, the response time measurements are conducted with various input signal frequencies ranging from 1 kHz to 20 MHz, with the corresponding rise/fall times and error bands shown in Figure 4d,f. Observed that the response time of the device is quite steady until the frequency of the input signal reaches about 100 kHz and then goes down slowly. The fastest response time is obtained at 20 MHz input signal, with a rise time of 9.02 ns and a fall time of 9.03 ns. The 20 MHz is the highest frequency currently employed in response speed testing, and there is no distortion in the output waveform of our devices in this high‐frequency range. The response times of typical NACDs constructed with various materials, including Si, Metal, nanotubes, and graphene are compared in Figure 4f.^[^ ^12^, ^19^, ^33^ ^]^ This highlights the fact that our GaN NACD offers response times as low as 9 ns, faster than previously reported for NACDs. Response speed can be improved further by reducing the device emission area and capacitance, as well as optimizing the electrode material and measurement setup.

### 2.5 Temperature‐Dependence of the Performance of GaN NACDs

**Figure 5:**
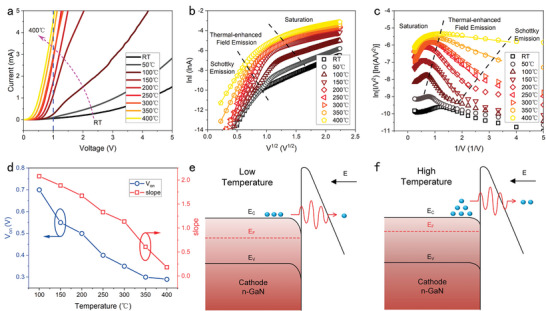
The output characteristics of the GaN NACDs (C1) under varying temperatures. a) *I–V* characteristics under the temperature from room temperature (RT) to 400 °C. b) *I*–*V* characteristics in Richardson–Schottky coordinates (ln*I* versus *V* ^1/2^) for different temperatures. Linearity in Richardson–Schottky coordinates defines Schottky emission at low voltages, followed by thermal‐enhanced field emission and saturation regions. c) Corresponding FN plots under varying temperatures. The linearity in FN coordinates determines the thermally assisted field emission and then follows the saturation region. d) *V*_on_ and slope under varying temperatures. e,f) Energy band schematic diagrams under low temperature (e) and high temperature (f), respectively. With increasing temperature, n‐type GaN has a higher Fermi level and a lower surface barrier height, allowing electrons to tunnel through a thinner barrier.

Because of their “junction‐less” construction, NACDs are considered competitive candidates for high‐temperature applications as compared to Si‐based solid‐state devices, which generally have a maximum operating temperature of 125 to 150 °C. Here, the temperature dependence of GaN NACDs performance is examined. Before testing, the devices were baked for 2 h on a heating board at 100 °C to remove water vapor and other surface contaminants. **Figure** **5**a depicts the *I–V* characteristics of the C1 device with a voltage range of 0 to 5 V, measured at temperatures ranging from room temperature (RT) to 400 °C with a 50 °C increment. As noted, the output current rises with increasing temperature, from 63.32 µA at RT to 4.58 mA at 400 °C with 1 V.

To investigate the mechanisms of the GaN NACD operating under high temperatures, the *I*–*V* characteristics of the device of C1 are plotted in Schottky and FN coordinates, as shown in Figure 5b,c. At low voltages, electron emission mainly follows Schottky emission. As the voltage increases at a certain temperature, the device's working mechanism transitions from Schottky emission to field emission. Furthermore, as illustrated by the left dashed line in Figure 5b, the transition voltage drops with increasing temperature. The possible reason for this is that the electrons occupy higher energy levels due to thermal effects, and when the temperature increases, the electrons can tunnel through the GaN–air barrier with a lower electric field, causing the mechanism transition from Schottky emission to field emission.

At high voltages (thermal‐enhanced field emission region in Figure 5c), linearities are observed in the FN coordinates, confirming the field emission mechanism in this region. Thermal‐enhanced field emission^[^ ^34^ ^]^ explains why the current increases with temperature and appears linear in the FN coordinate. When the voltage is further increased, the saturation region of the device characteristics appears (see saturation region in Figure 5c). The space charge effect^[^ ^35^ ^]^ is responsible for the behavior of positive linearity in FN coordinates. The charges in the air channel build with increasing voltage, which in turn reduces the electric field at the cathode surface and limits the emission current for a given voltage.^[^ ^35a^ ^]^ As the temperature increases, the voltage transition from thermal‐enhanced field emission to saturation becomes smaller, as shown by the left dashed line in Figure 5c. As the temperature rises, electrons tunnel through the thinner barrier and generate more space charge in the air channel, thereby entering the saturation mechanism at lower voltages.

Figure 5d shows the *V*_on_ and slope extracted from the FN coordinates. The *V*_on_ drops with increasing temperature, from 0.70 V at 100 °C to 0.29 V at 400 °C, and the slope decreases from 2.078 to 0.182. According to Equation (1), the slope depends on both *φ* and *β*. Consequently, the value of the slope decreases as the temperature rises, which is probably due to a decrease in *φ*, an increase in *β*, or a combination of both. The increase of *β* may be caused by temperature‐dependent desorption of contaminants from the electrode surface, changing the roughness of the GaN surface.^[^ ^36^ ^]^ As the temperature increases, the Fermi level gradually rises,^[^ ^37^ ^]^ causing a decrease in the work function. Also, temperature‐dependent oxygen molecule adsorption^[^ ^38^ ^]^ could reduce the work function. Moreover, the decline in surface barrier height^[^ ^39^ ^]^ with increasing temperature permits electrons to tunnel through thinner barriers to generate large field emission currents. Figures 5e,f show the energy band diagrams at low and high temperatures. At high temperatures, both the temperature‐induced elevation of the Fermi level and the height of the surface barrier decrease, permitting electrons on the GaN surface to tunnel through the thinner interfacial barrier. This property accounts well for the reported thermal‐enhanced effect of GaN NACD field emission, where the apparent reduction in *V*_on_ and marked improvement of emission current result from the temperature‐dependent decline in *φ* and increment in *β*.

## 3 Conclusions

In summary, we proposed a vertical GaN NACD with an air channel down to 50 nm fabricated by low‐cost, IC‐compatible manufacturing technologies on a 2‐inch sapphire wafer, rather than expensive and complicated processes such as EBL and FIB. Record, our NACD exhibits an output current of over 10 mA at 10 V with a turn‐on voltage of only a few volts, which is 2–3 orders of magnitude higher than previously reported results. Additionally, the device exhibits improved stability in three working modes, including cycle, long‐term, and pulsed voltage testing. It also has a fast response time of fewer than 10 ns. The advantages of our devices are attributed to both the excellent properties of GaN and the robust IC‐compatible process used to create the vertical device structure, which avoids complex and delicate design. These GaN NACDs have the potential to play an essential role in future electronic devices, providing valuable insight into the realization of practical NACTs. Further research into the operation mechanism and the effect of anode size and temperature on the static/temporal characteristics of the device will enhance the comprehensive understanding of NACTs.

Future heterostructure‐based GaN NACT architecture concepts^[^ ^40^ ^]^ have significant research potential. Modifying the energy band through components, doping, defects, etc. can allow for greater electron emission freedom. While much progress has been made in improving the static characteristics of NACTs, research into their dynamic and high‐frequency properties remains limited. It will be crucial in the future to explore ways to minimize parasitic effects while preserving high‐frequency dynamic features and ensuring adequate output current and other properties.

## 4 Experimental Section

### Device Fabrication

The GaN NVCDs were manufactured with IC‐compatible techniques, including plasma‐enhanced chemical vapor deposition (PECVD), photolithography, electron beam evaporation, lift‐off, and BOE wet‐etching process. Step‐by‐step fabrication details of the proposed GaN NACDs are provided in Figure S4, Supporting Information. The starting material was a 4.5 µm thickness layer of n‐GaN (Si: 3×10^18^ cm^−3^) produced on *c*‐sapphire by metal organic chemical vapor deposition. Initially, the oxide layer was deposited using PECVD in a mixture of SiH_4_ and N_2_O. By modifying the deposition parameters of PECVD, the thickness of the oxide layer may be precisely adjusted. Here, a 50 nm SiO_2_ layer was deposited in 80 s on a GaN wafer. The substrate was then patterned using photolithography, and the residual photoresist on the pattern region was removed by O_2_ plasma of 30 W for 30 s. Next, 10/120 nm Ti/Au electrodes with Ti as the adhesion layer were deposited, followed by a lift‐off process to generate a multilayer structure of Au/SiO2/GaN. A BOE wet etching (NH_4_F:HF = 6:1) process was then employed to remove the SiO_2_ under the anode edge, creating an air channel between the Au and GaN. BOE wet etching time permitted precise control over the size of the air channel.^[^ ^19^ ^]^ Figure S5, Supporting Information demonstrates the electrical performance of C1 devices with various BOE wet etching durations (40, 60, and 80 s). Figure S6, Supporting Information demonstrates the manufacturing scalability of GaN NACDs.

A FIB system (Helios G4 UX) was applied for cutting the device to obtain the cross‐sectional profile. The acceleration voltage of Ga^+^ was 30 keV. For this work, an ion‐beam current of 20 nA was utilized.

### Device Performance Measurement

Current–voltage (*I–V*) characteristics of GaN NACDs were measured using a digital source meter (Agilent 2450 SMU). In addition, the *I–V* data shown was the average of five tests unless stated. The square wave voltage was generated by a signal generator (Tektronix AFG31152) and the output voltage was recorded by an oscilloscope (Tektronix MDO3104). All experiments were conducted under ambient conditions.

### Multiphysics Simulation

The commercial finite element method software (COMSOL Multiphysics 5.6) was used to simulate the potential distribution in the air channel and the deformation of the Au electrode under high electric field intensity. The electrostatic module and solid mechanics module were employed to solve for the potential distribution and the Au electrode deformation, respectively. The material parameters including the dielectric constant and Young's modulus were adopted as default values from the COMSOL material library.

## Conflict of Interest

The authors declare no conflict of interest.

## Supporting information

Supporting Information

## Data Availability

The data that support the findings of this study are available from the corresponding author upon reasonable request.
