# Supplementary material for: Fast Response GaN Nanoscale Air Channel Diodes with Highly Stable 10 mA Output Current toward Wafer‐Scale Fabrication
Source: Adv Sci (Weinh). 2023 Apr 20;10(17):2206385. doi: 10.1002/advs.202206385 (PMC10265105; doi:10.1002/advs.202206385)
Supplement: Supplementary file 1 — Supporting Information [file ADVS-10-2206385-s001.pdf]

ADVANCED SCIENCE
Open Access

Supporting Information

for *Adv. Sci.*, DOI 10.1002/advs.202206385

Fast Response GaN Nanoscale Air Channel Diodes with Highly Stable 10 mA Output Current toward Wafer-Scale Fabrication

Yazhou Wei, Feiliang Chen, Ruihan Huang, Jianpeng Zhao, Haiquan Zhao, Jiachao Wang, Mo Li and Jian Zhang*

Supporting Information

Fast response GaN Nanoscale Air Channel Diodes with Highly Stable 10 mA Output Current Toward Wafer-scale Fabrication

Yazhou Wei, Feiliang Chen, Ruihan Huang, Jianpeng Zhao, Haiquan Zhao, Jiachao Wang, Mo Li, Jian Zhang*

1. Leakage tests of the devices

Figure S1 illustrates the *I-V* characteristics of potential leakage paths for GaN NACDs to rule out the devices operating in leakage mode. Figure S1a presents the results of three leakage tests between two different top Au cathodes. We observe the leakage current is less than 30 pA@10 V between the two Au electrodes, confirming the absence of Au bending to contact with GaN and the excellent insulating properties of SiO_2 between the Au electrodes and GaN. Figure S1b shows the results of three leakage tests between two different bottom Au electrodes. Note that the contact current between the two bottom electrodes is less than 500 μA@10 V which is much lower than the current during the device operation (*I* > 10 mA). Leakage tests of the vertical Au/SiO_2(50nm)/Au MIM structures are displayed in Figure S1c, with a maximum leakage current of sub 300 pA@10 V, indicating that the SiO_2 deposited by PECVD has good insulation properties. These results confirm that the device conducts electron transport through the air channel rather than the conduction mechanism formed by the contact between the Au electrode and the GaN.

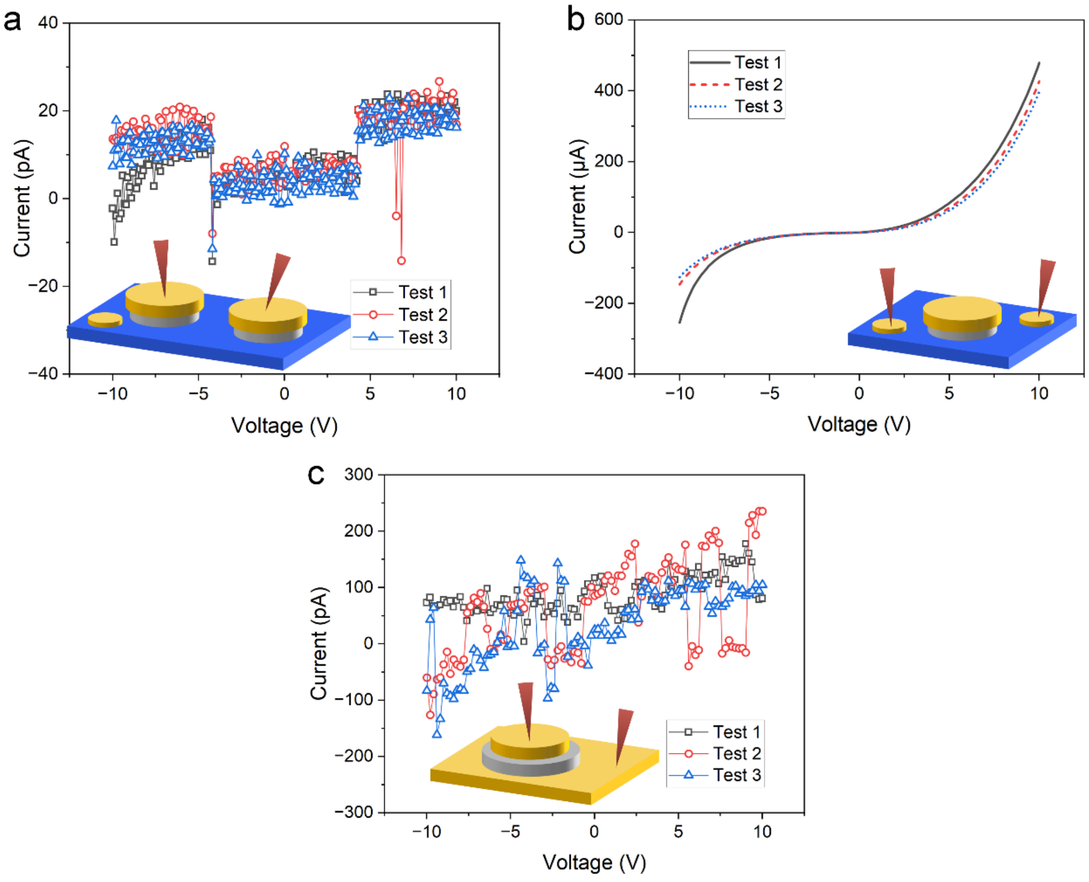

Figure S1. Leakage tests of devices. a Leakage test between any two top Au electrodes. **b** Leakage tests between any two bottom electrodes. **c** Leakage tests of the vertical Au/SiO_2/Au MIM structure.

2. Schottky emission and field emission theory

2.1 Schottky emission theory

Schottky emission is a process of electron emission through field-enhanced thermal activation[1]. The expression for Schottky emission related to electric field (*E*) and temperature (*T*) is[2]

$$J = AT^2 \exp\left(\varphi - \frac{\beta E^{\frac{1}{2}}}{kT}\right) \tag{1}$$

where *J* is the Schottky emission current density, *T* is the absolute temperature, *A* is the Richardson's constant, *φ* is the work function of the cathode, *β* is the relative dielectric constant of the cathode, *k* is the Boltzmann's constant, and *E* is the applied electric field. From equation (1), at a constant temperature, the linear relationship between ln*J* and $E^{1/2}$ indicates Schottky emission.

2.2 Field emission theory

FN tunneling is a quantum mechanism where the electron wave function penetrates the barrier when it becomes thin enough. The current density of the field emission is expressed as[3]

$$J = \frac{e^3 F^2}{8\pi h\varphi t(y)} \exp\left[-\frac{8\pi (2m)^{1/2} \varphi^{3/2}}{3heF} v(y)\right] \tag{2}$$

where *J* is the field emission current density, *e* and *m* are the charge and mass of the electron, respectively, *h* is the Planck's constant, *F* is the cathode surface electric field, *φ* is the work function of the cathode, *y* is a parametric function of *F* and *φ*, *t*(*y*) and *v*(*y*) are approximated constants. Further, the FN equation can be simplified to the equation of *I* and *V*, stated as[4]

$$I = AV^2 \exp\left(-\frac{B}{V}\right) \tag{3}$$

where A and B in equation (3) are

$$A = 1.54 \times 10^{-6} \beta^{2} \varphi^{-1} d^{-2} \alpha \quad (4)$$

$$B = 6.83 \times 10^{9} d \varphi^{3/2} \beta \quad (5)$$

where α is the field emission area and β is the field enhancement factor. The logarithm of both sides of Equation (3) yields the following linear relationship between $\ln(I/V^2)$ and $1/V$:

$$\ln\left(I/V^{2}\right) = \ln A - B/V \quad (6)$$

3. Electric field distribution in the air channel and the deformation of the Au electrode

The electric field and potential distribution in the air channel are depicted in Figure S2a. A 2-D finite element method (software: COMSOL Multiphysics 5.6) is utilized to numerically calculate the electric field distribution between the bent Au anode and GaN cathode with a 10 V voltage. The electric field of the GaN surface facing the Au electrode is about 2×10^8 V/m and decays rapidly outside the facing region. Therefore, the main emission zone is the GaN surface facing the Au electrode.

Finite element simulations were carried out to investigate the deformation of Au electrodes since Au is soft and deformable, especially under high electric field, as illustrated in Figure S2b. The default parameters of the COMSOL material library were utilized as the material parameters for the simulation, including the dielectric constant and the Young's modulus. Actual structural parameters of the devices were adopted in the simulation, such as the thickness of Ti, Au and SiO_2 layer is 10 nm, 120 nm, and 50 nm, respectively; the depth of the air channel is 300 nm. It can be noted that the deformation at the edge of the Au electrode is only 0.036 nm at 10 V bias, with no significant bending. Additionally, as shown in Figure S2c, we investigated the deformation of an Au electrode with varied air channel depths (300 nm to 1000 nm) at various voltages (2 V to 20 V). Note that the 1000 nm depth air channel has significant deformation (20 nm) at higher voltages (20 V), which should be avoided in device operation.

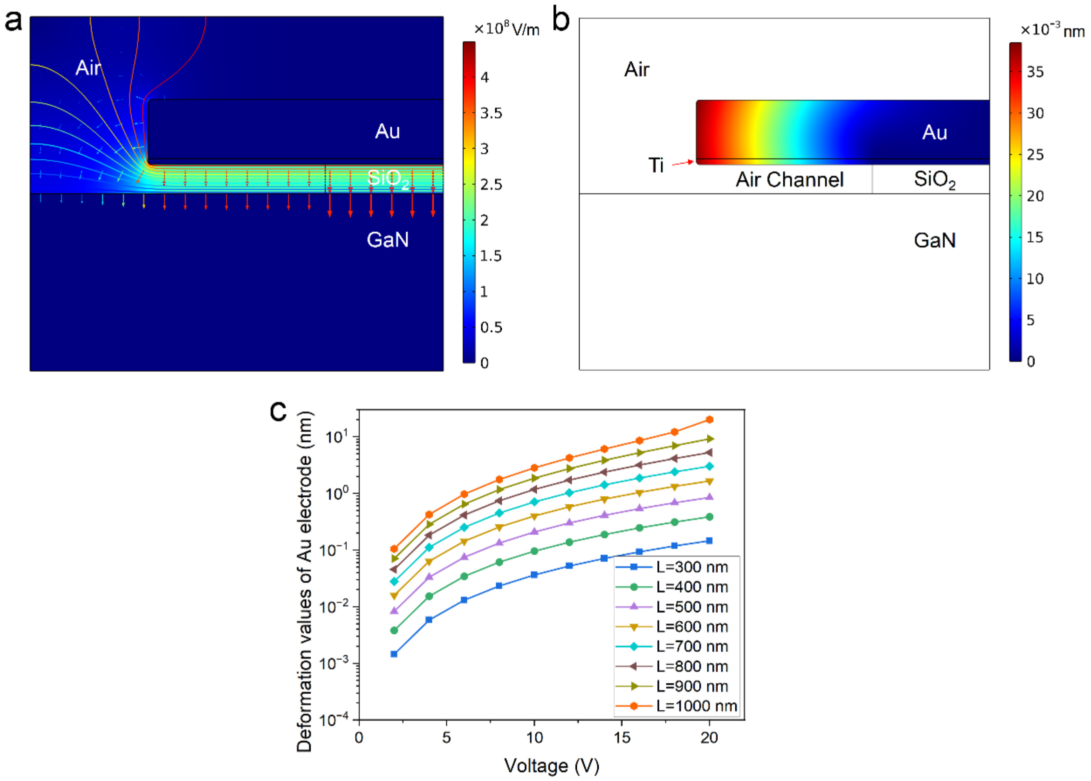

Figure S2. Electric field distribution and Au electrode deformation. a Electric field distribution in the air channel with an anode voltage of 10 V. **b** The deformation of the Au electrode at a potential of 10 V for GaN. **c** The deformation of Au electrode with various air channel depths (300 nm to 1000 nm) at various voltages (2 V to 20 V).

4. Cross-sectional SEM images of the GaN NACDs

The cross-sectional shape of the devices C1-C4 after BOE etching was obtained by Focused Ion Beam (FIB), as shown in Figure S3. C1-C4 devices were prepared on the same substrates with the same preparation conditions and BOE wet etching times. For C1 to C4, all devices have a dielectric layer (SiO_2) thickness of 50 nm and air channel depths of 300 nm, 300 nm, 290 nm, and 310 nm. Their same thickness of dielectric layer and near-identical depth of channel highlight the good reliability and consistency of our preparation process, which is essential for the fabrication of GaN NACDs.

The deformation of the Au electrode edges was observed after FIB cutting with a high-energy ion beam at 30 keV. The FIB with Ga^+ ions at keV can introduce nanoscale tensile stresses in materials such as Al and Au, which has previously been employed in several reports for its capacity to deform two-dimensional films into three-dimensional structures.[5] Consequently, the bombardment of the high-energy ion beam during the FIB process and the lack of support from the dielectric layer of the electrode after the BOE etching, contributed to the bending of the Au electrode edges. Whereas the channel of the device by BOE etching is uniform and stable, as can be demonstrated by the SEM image in Figure 1c. Moreover, as shown in the section 3 of Supporting Information, the deformation of the Au electrodes during device operation is negligible.

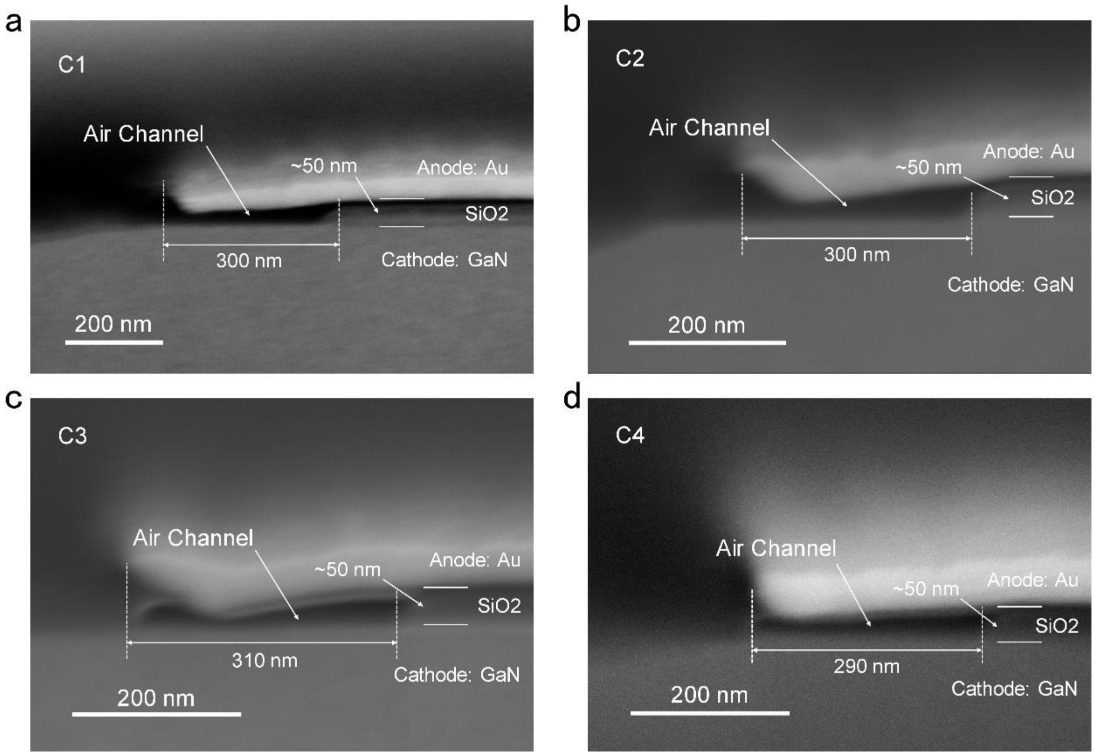

Figure S3. Cross-sectional SEM images of the edges and air channels of C1 (a), C2 (b), C3 (c), and C4 (d).

5. The fabrication process of proposed GaN NACDs

The proposed GaN NACDs' step-by-step preparation techniques are depicted in Figure S4. To begin, the oxide layer was created using plasma-enhanced chemical vapor deposition (PECVD) in a gas combination of SiH_4 and N_2O. The substrate was then patterned by photolithography and cleaned with O_2 plasma to remove any remaining photoresist from the pattern region (Figure S4a). Following that, 10/120 nm Ti/Au electrodes with Ti as the adhesion layer were deposited (Figure S4b), followed by a lift-off process to generate a multilayer structure of Au/SiO_2/GaN (Figure S4c). The local SiO_2 was subsequently removed using a BOE wet etching method, forming an air channel between the Au and GaN (Figure S4d).

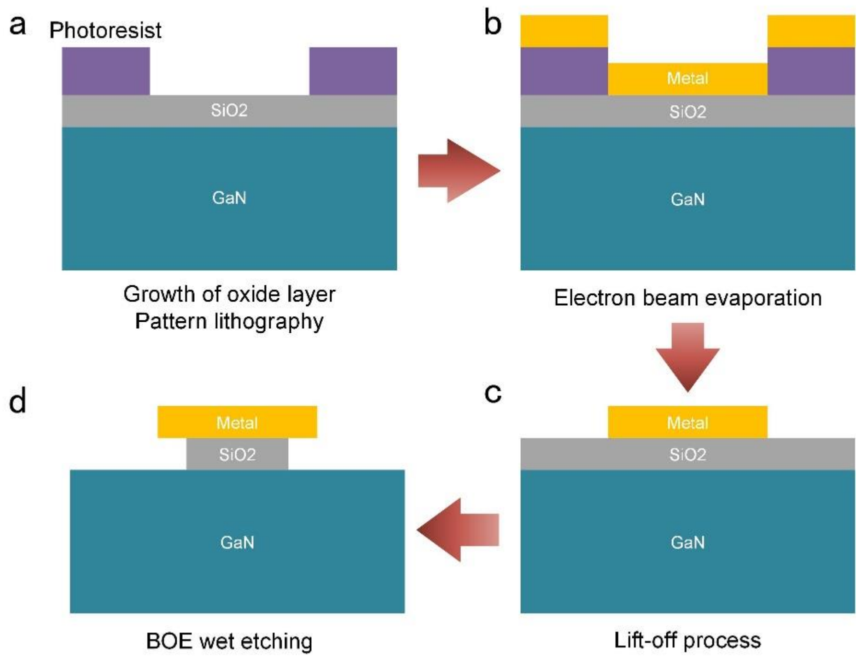

Figure S4. The fabrication processes of the proposed GaN NACDs.

6. Electrical characteristics of the GaN NACDs with different BOE wet etching durations

We exhibit the electrical performance of C1 with different BOE wet etching durations (40 s, 60 s, and 80 s) in Figure S5, including *I*-*V* characteristics and FN plots. As demonstrated in Figure S5a, extending the etching time increases the field emission current of GaN and Au, with GaN having a larger field emission current than Au. The BOE wet etching is responsible to realize the air channel. Longer etching times result in deeper air channels and larger emission areas, resulting in stronger field emission currents. Furthermore, the lower electron affinity of GaN compared to the work function of Au and the asymmetric device structure allow for a greater field emission current of the GaN cathode, resulting in the device's rectification behavior. Furthermore, the FN plots of Au and GaN cathodes for various etching durations, as shown in Figure S5b and Figure S5c, demonstrate that the device's working mechanism is field emission at V>2 V.

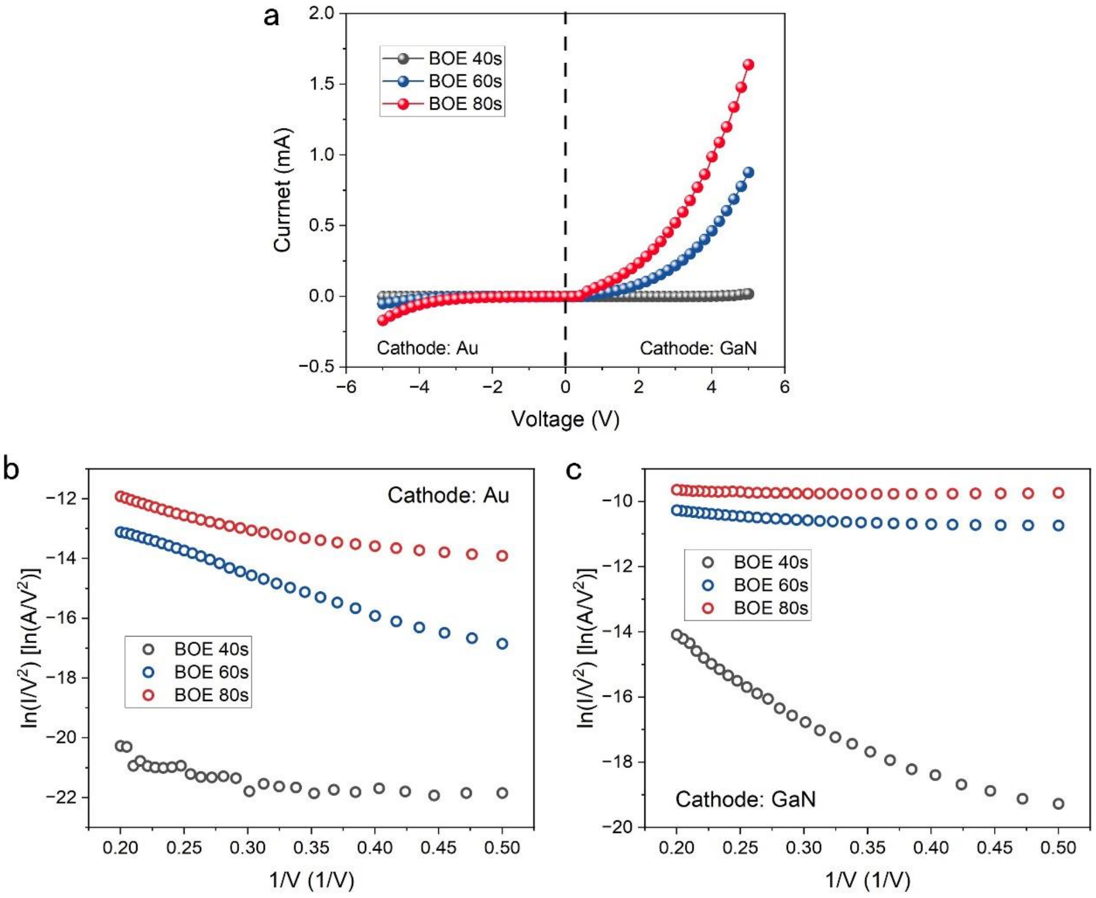

Figure S5. ***I-V*** **characteristics and corresponding FN plots of GaN NACD with various BOE wet etching durations. a** *I-V* characteristic curves of GaN NACDs with different BOE wet etching durations (40 s, 60 s, and 80 s). **b, c** FN plots of the GaN NACD with Au (**b**) and GaN (**c**) as cathodes, respectively.

7. Manufacturing scalability of GaN NACDs

Figure S6a shows a fabricated 2-inch sapphire wafer with 168 devices and a density of 8.23 units/cm^2, showing the feasibility of wafer-scale fabrication of GaN NACDs. The device density is highly dependent on the design of the structure and the wafer layout, with further optimization, it is possible to fabricate even more GaN NACDs on 2-inch substrates in the future. Figure S6b shows dozens of devices in a 3×2 cm^2 area, including electrodes of various sizes and different shapes. A top-view microscope image of a single device is shown in Figure S6c. Figure S6d and Figure S6e present top-view SEM images of square and circle electrode edges with partial magnification, respectively.

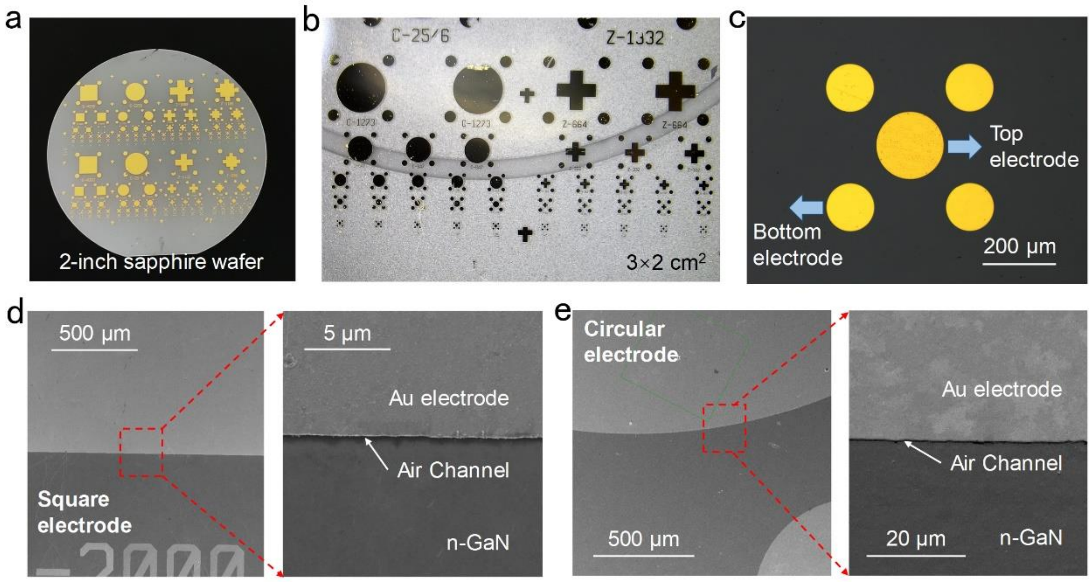

Figure S6. Images of manufactured GaN NACDs. a A prepared 2-inch sapphire wafer. **b** Various sizes and shapes of devices on a 3×2 cm^2 area. **c** Top-view microscope image of a single device. **d, e** Top view SEM images of square (d) and circular electrode edges (e) and their partial enlargement.

Reference

[1] S. Nirantar, T. Ahmed, M. Bhaskaran, J. W. Han, S. Walia, S. Sriram, *Adv. Intell. Syst.* **2019**, 1, 1900039.

[2] Y. H. Sun, D. A. Jaffray, J. T. W. Yeow, *Carbon* **2013**, 58, 87.

[3] R. H. Fowler, L. Nordheim, *Proc. R. Soc. London, Ser. A* **1928**, 119, 173.

[4] J. W. Han, J. S. Oh, M. Meyyappan, *IEEE Trans. Nanotechnol.* **2014**, 13, 464.

[5] a)K. Chalapat, N. Chekurov, H. Jiang, J. Li, B. Parviz, G. S. Paraoanu, *Adv. Mater.* **2013**, 25, 91; b)A. J. Cui, Z. Liu, J. F. Li, T. H. H. Shen, X. X. Xia, Z. Y. Li, Z. J. Gong, H. Q. Li, B. L. Wang, J. J. Li, H. F. Yang, W. X. Li, C. Z. Gu, *Light-Science & Applications* **2015**, 4, e308; c)L. Xia, W. G. Wu, J. Xu, Y. L. Hao, Y. Y. Wang, Ieee, presented at *19th IEEE International Conference on Micro Electro Mechanical Systems (MEMS 2006)*, Istanbul, TURKEY, Jan 22-26, **2006**.
